# Paratesticular Embryonal Rhabdomyosarcoma Masquerading as Epididymitis: A Diagnostic Challenge in Adolescents

**DOI:** 10.1002/ccr3.73219

**Published:** 2026-07-31

**Authors:** Saif Khaled Abdalhadi Azzam, Lina Barhoum Barhoum, Ammir Abuzahra, Alaa Alazzeh, Khader Hassouneh, Nour Aldin K. Fakhory, Mohd Othman Lafi, M. I. Jawabreh Issa, Ibrahim Alzatari, Hamza Zughaier

**Affiliations:** ^1^ Department of Clinical Medical Sciences, Faculty of Medicine and Health Sciences Palestine Polytechnic University Hebron West Bank Palestine; ^2^ College of Medicine, Hebron University Hebron West Bank Palestine; ^3^ Radiology Department Al‐Ahli Hospital Hebron West Bank Palestine; ^4^ Department of Pathology Al‐Ahli Hospital Hebron West Bank Palestine

**Keywords:** case report, embryonal rhabdomyosarcoma, epididymitis, paratesticular rhabdomyosarcoma, pediatric oncology, scrotal mass

## Abstract

Paratesticular rhabdomyosarcoma is a rare malignancy in children and adolescents that frequently presents diagnostic challenges when mimicking benign inflammatory conditions. We report the case of a 14‐year‐old previously healthy male who presented with acute right scrotal pain and swelling initially diagnosed as epididymo‐orchitis. Despite 1 month of intensive antibiotic therapy including intravenous meropenem, the patient showed no clinical improvement. Serial ultrasound examinations demonstrated a heterogeneous paratesticular mass with increased vascularity, prompting MRI evaluation that revealed an 8 × 6.4 × 9.5 cm heterogeneous enhancing mass. CT staging showed prominent para‐aortic and inguinal lymph nodes without distant metastases. The patient underwent radical inguinal orchiectomy, and histopathology confirmed embryonal rhabdomyosarcoma with spindle cell features and focal anaplasia. Immunohistochemistry demonstrated positive staining for desmin and myogenin, confirming skeletal muscle differentiation. The patient was referred for multimodal chemotherapy according to Children's Oncology Group protocols. This case highlights the critical importance of maintaining high clinical suspicion for paratesticular malignancy when pediatric scrotal masses fail to respond to appropriate antibiotic therapy, as diagnostic delays can significantly impact staging and treatment outcomes.

## Key Clinical Message

Paratesticular rhabdomyosarcoma frequently masquerades as benign epididymitis in adolescents. High clinical suspicion is essential when pediatric scrotal masses fail to respond to appropriate antibiotics, as delayed diagnosis significantly impacts outcomes.

## 1 Introduction

Rhabdomyosarcoma (RMS) is the most common soft tissue sarcoma in children and adolescents, accounting for approximately 50% of all soft tissue sarcomas in this age group, with an annual incidence of 4.6 cases per 1 million children younger than 20 years [1]. Paratesticular RMS, while rare, represents approximately 7% of all RMS cases and is the most common malignant paratesticular tumor in the pediatric population, with two incidence peaks occurring between ages 2–6 and 15–19 years [2]. The embryonal subtype comprises 90%–97% of paratesticular RMS cases, generally conferring a favorable prognosis compared to other RMS subtypes [1]. With contemporary multimodal therapy, 5‐year overall survival for localized paratesticular RMS exceeds 94%, making early diagnosis and appropriate management critical [3].

A significant challenge in diagnosing paratesticular RMS is its propensity to mimic benign inflammatory conditions. When presenting with painful scrotal swelling and signs of inflammation, paratesticular RMS is frequently misdiagnosed as epididymo‐orchitis, leading to delayed diagnosis and inappropriate antimicrobial therapy [4]. This diagnostic pitfall carries significant clinical consequences, as delayed diagnosis is associated with larger tumors at presentation, increased risk of retroperitoneal lymph node involvement (which occurs in up to 40% of patients), and compromised surgical margins, all of which negatively impact outcomes [5]. Furthermore, the use of adult‐directed treatment protocols rather than pediatric‐adapted Children's Oncology Group (COG) regimens in adolescents with paratesticular RMS has been associated with catastrophic treatment failures and early metastatic relapse, even in cases initially presenting with completely resectable disease and favorable prognosis [6].

We report an adolescent with paratesticular embryonal RMS presenting with acute painful scrotal swelling initially managed as epididymitis. This case highlights the importance of early recognition of atypical imaging findings and failure of antibiotic therapy as indications for prompt evaluation for underlying malignancy.

## 2 Case History/Examination

A 14‐year‐old previously healthy male presented with a 2‐day history of acute right scrotal pain and rapidly worsening swelling. Although the painful enlargement was acute, it is possible that the underlying paratesticular mass had been present but clinically unnoticed before secondary inflammation developed. The swelling began small and progressively enlarged, accompanied by aching scrotal pain rated 10/10 in intensity that worsened with movement. The patient denied fever, vomiting, dysuria, abdominal pain, or bowel changes. His medical and surgical histories were unremarkable, and he was taking no regular medications.

On physical examination, the right hemiscrotum was significantly enlarged and tender. The left testis was clinically normal. Initial scrotal Doppler ultrasound revealed a large, heterogeneous paratesticular mass measuring 5 × 5 cm adjacent to the right testicle with increased internal vascularity on Doppler imaging. The right epididymis was markedly enlarged (5.1 × 3.5 cm) with heterogeneous echotexture and increased vascularity, associated with mild pyocele and right scrotal skin thickening up to 8 mm. The initial radiological impression was severe right epididymitis with phlegmon formation and pyocele. The atypical imaging finding of internal vascularity within the mass, however, raised concern for an underlying neoplastic process rather than simple inflammation, prompting close radiological follow‐up.

**IMAGE 1:**
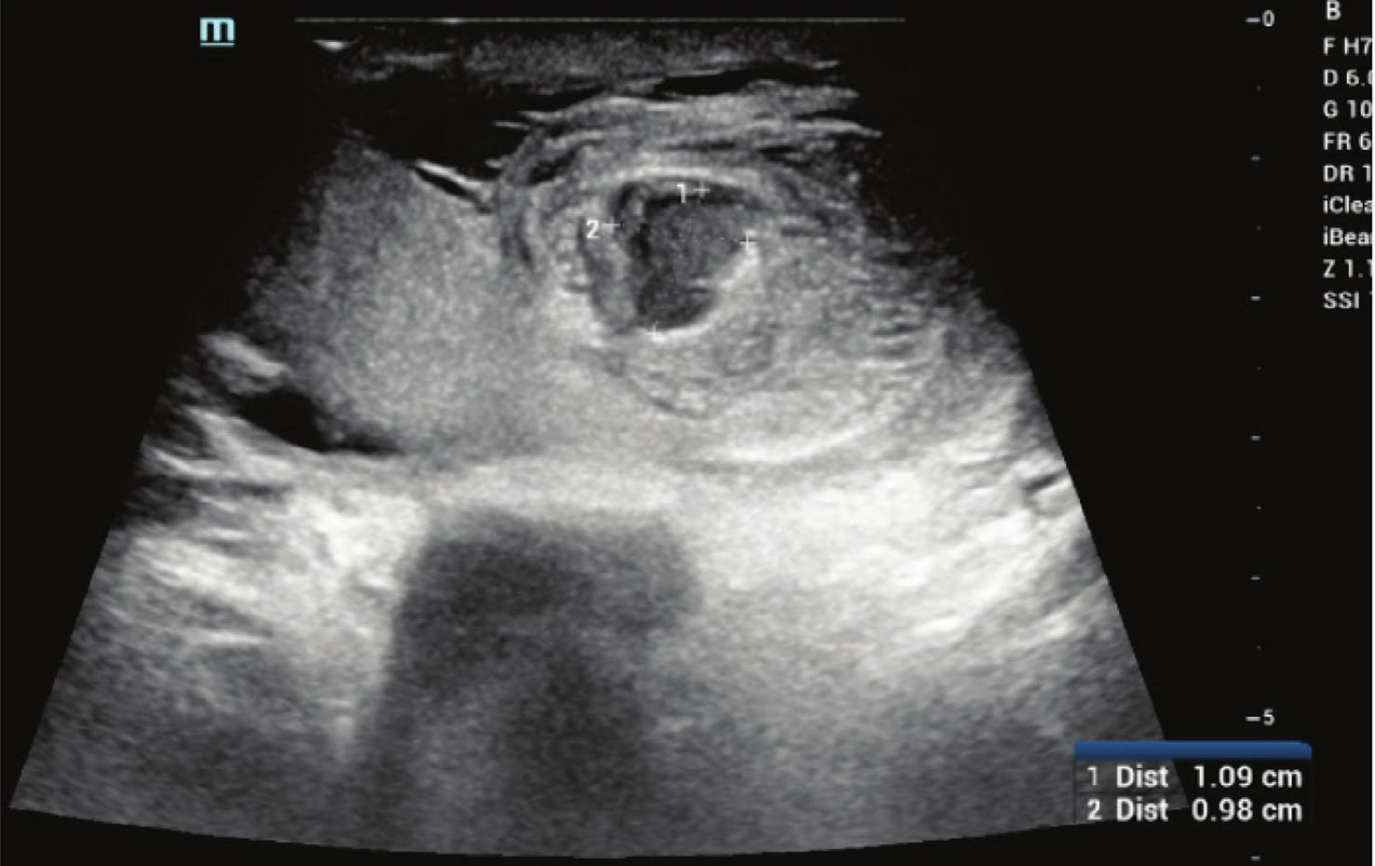
Ultrasound shows scrotal ultrasound demonstrates a normal right testicle (arrow) with preserved echotexture.

**IMAGE 2:**
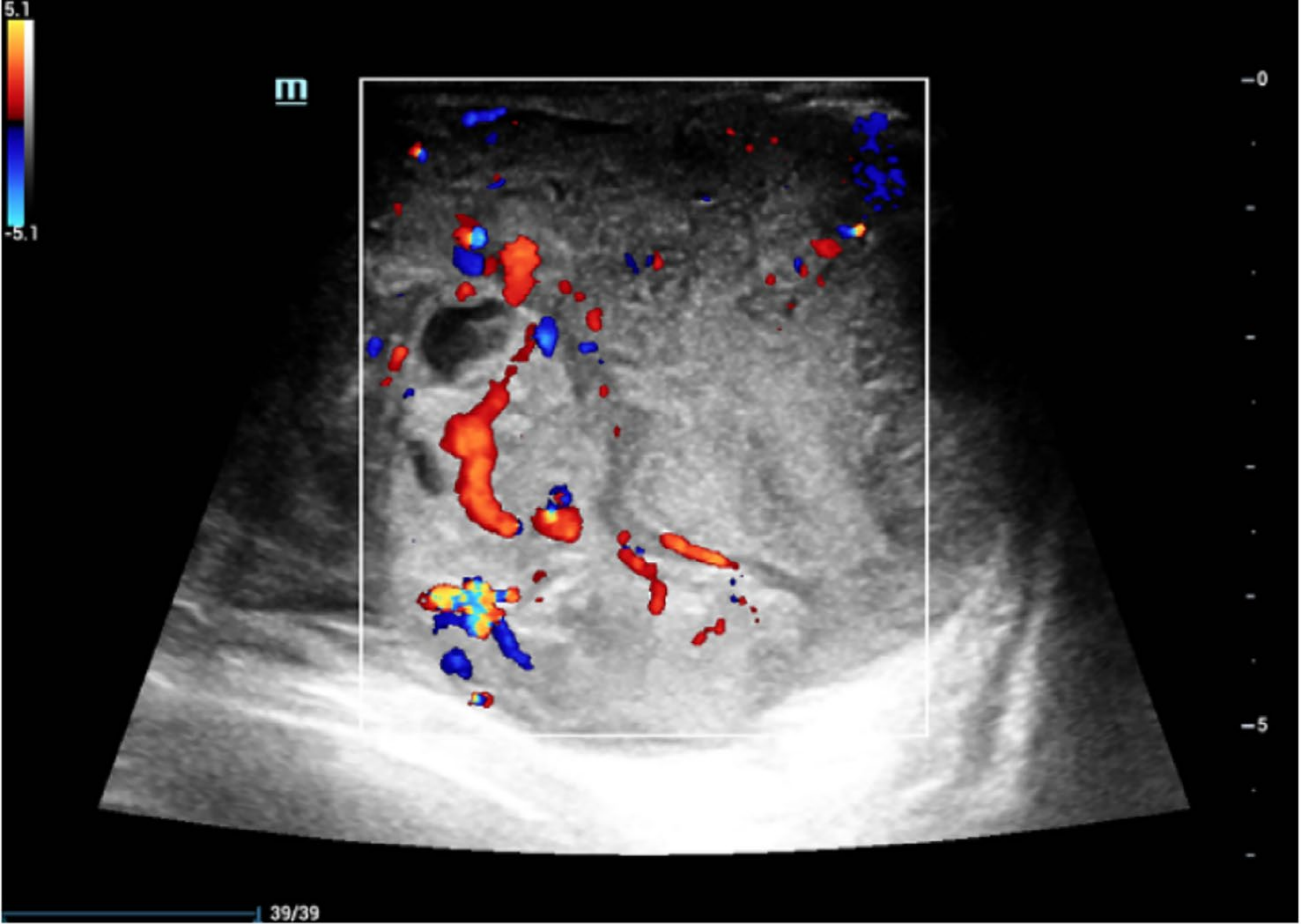
Color Doppler imaging demonstrates internal vascularity within the mass, a finding atypical for simple inflammatory enlargement and concerning for a follow up to R/O a neoplastic process.

Initial scrotal ultrasound demonstrates a normal right testicle (arrow) with preserved echotexture (Image 1). There is a heterogeneous right paratesticular mass with increased internal vascularity on color Doppler imaging, a finding atypical for simple inflammatory enlargement and concerning for a neoplastic process (Image 2).

The patient was treated for severe acute right epididymo‐orchitis with oral and intravenous antibiotics, including a 12‐day course of meropenem. Despite 1 month of intensive antibiotic therapy, there was minimal clinical improvement, with persistent right hemiscrotum enlargement. Serial ultrasound examinations documented persistent epididymal enlargement (5.5 × 4 cm) with heterogeneous echotexture, marked vascularity, and a small hypoechoic focus (1.2 × 1.1 cm) suggestive of abscess formation. The failure to improve with medical management and the imaging features concerning for an underlying mass necessitated advanced imaging evaluation.

Because the patient remained clinically stable and serial ultrasonography continued to favor an inflammatory process with possible abscess formation, conservative treatment was initially continued. However, persistent symptoms and the absence of meaningful clinical improvement despite prolonged antibiotic therapy prompted further evaluation with MRI.

Scrotal MRI was performed for improved tissue characterization and revealed a heterogeneous, enhancing paratesticular mass measuring 8 × 6.4 × 9.5 cm extending into the inguinal canal. On coronal T2‐weighted imaging, a lobulated, heterogeneous soft‐tissue mass was identified arising from the right epididymis with intermediate to high T2 signal intensity and internal areas of heterogeneity consistent with necrosis or myxoid components. The right testicle was displaced inferiorly and medially but remained separate and preserved with normal T2 signal intensity, supporting a paratesticular origin. On sagittal T1‐weighted pre‐contrast imaging, the mass appeared iso‐ to mildly hypointense to skeletal muscle. Following gadolinium administration, the lesion demonstrated heterogeneous enhancement with avid enhancement of solid components and non‐enhancing areas consistent with necrosis. Coronal and axial post‐contrast images confirmed clear delineation of the enhancing paratesticular mass from the normally enhancing right testicle, with irregular but well‐defined margins and no definite testicular invasion. The mass extended superiorly with mass effect on adjacent penile structures but no definite evidence of direct penile invasion.

**IMAGE 3:**
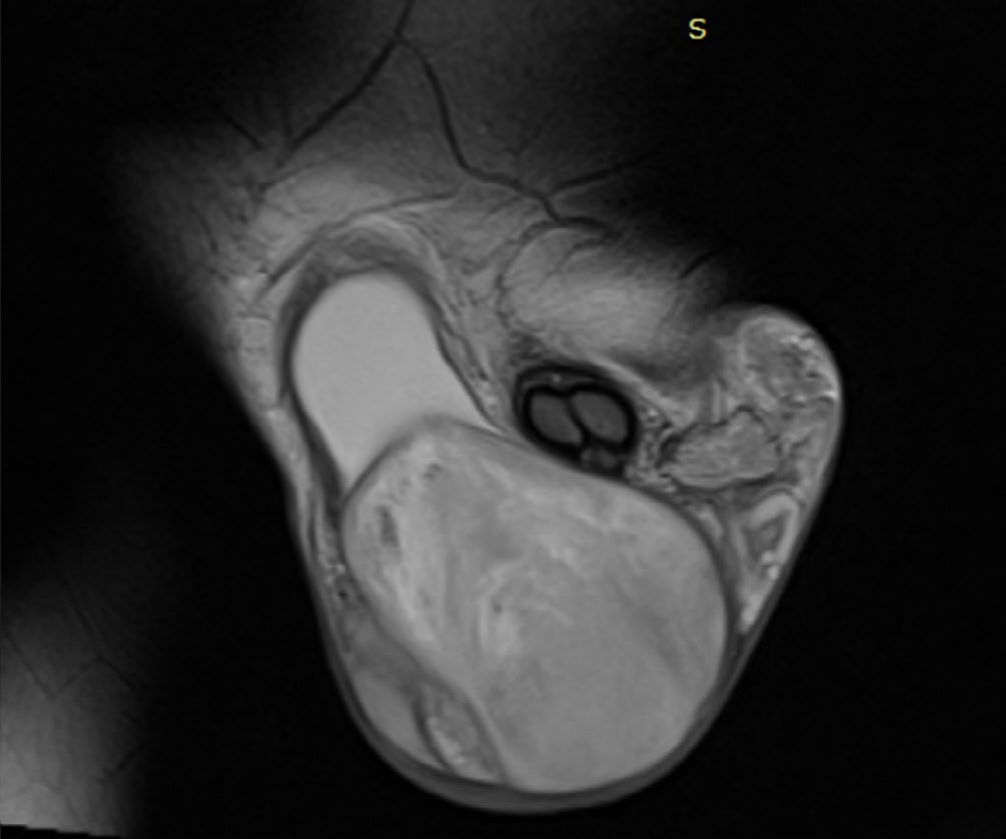
Coronal T2‐weighted image: There is a lobulated, heterogeneous soft‐tissue mass centered in the right paratesticular region, predominantly arising at the expected site of the right epididymis. The lesion demonstrates predominantly intermediate to high T2 signal intensity, with internal areas of relative heterogeneity, suggestive of necrosis or myxoid components. The mass causes mass effect on adjacent structures without clear invasion of surrounding tissues on this sequence.

**IMAGE 4:**
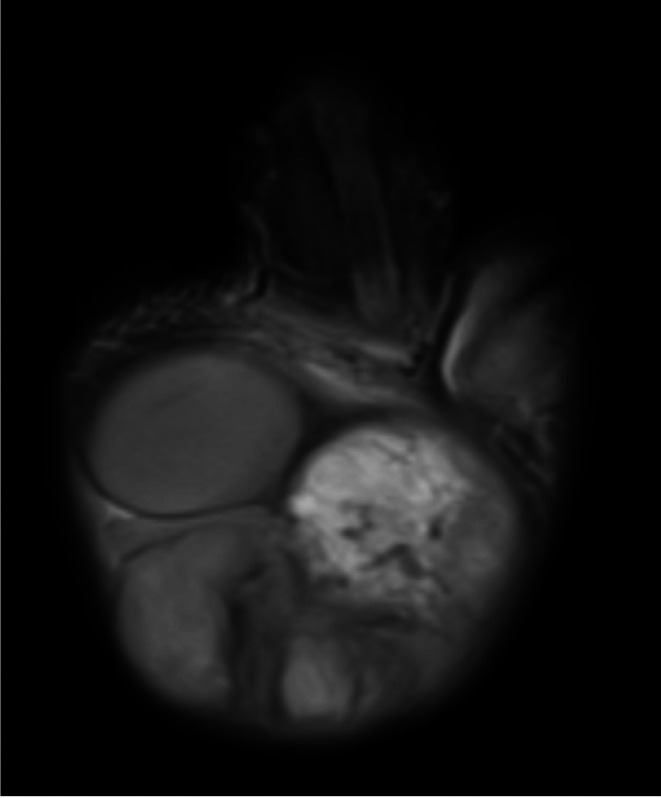
Coronal T2‐weighted image (with right testicle visualized): The right testicle (arrow) is displaced inferiorly and medially by the mass but appears separate and preserved, maintaining normal T2 signal intensity. This finding supports a paratesticular rather than intratesticular origin of the lesion.

**IMAGE 5:**
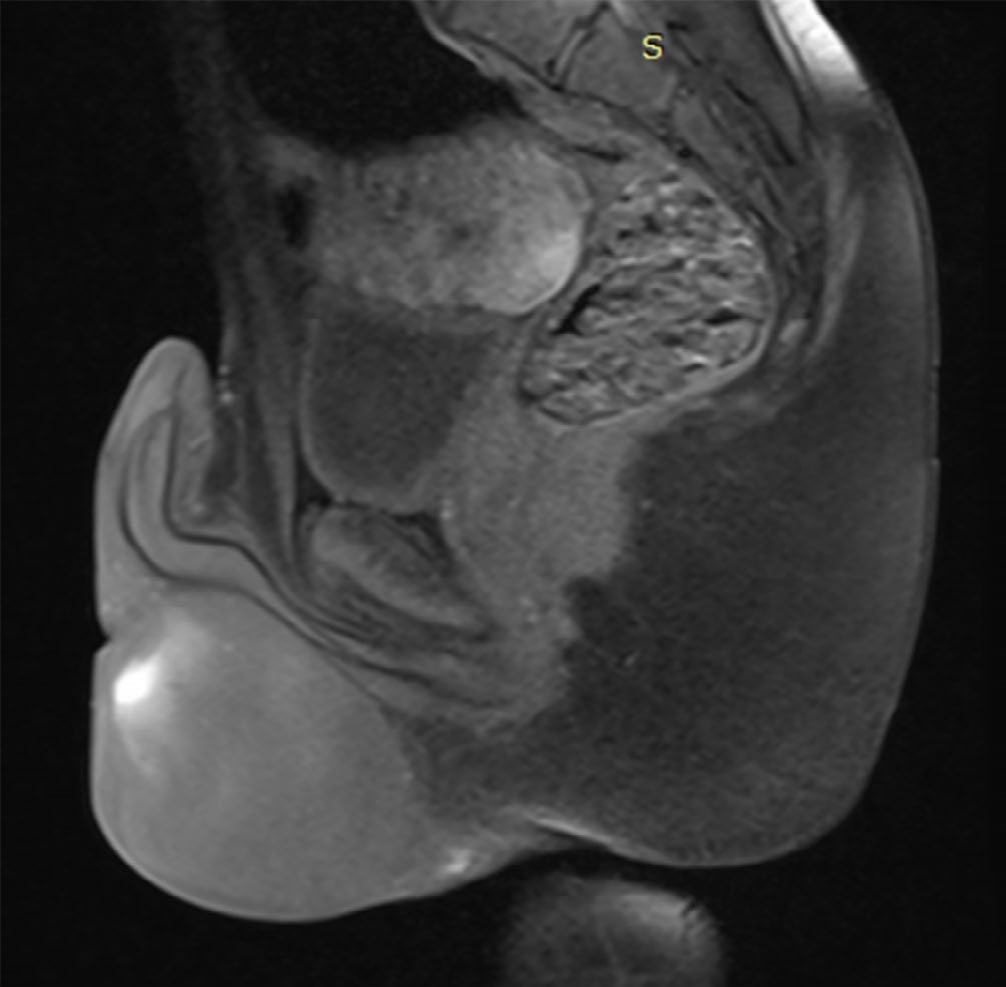
Sagittal T1‐weighted pre‐contrast image: The mass appears iso‐ to mildly hypointense relative to skeletal muscle on T1‐weighted imaging. No areas of intrinsic high T1 signal are identified to suggest hemorrhage or fat content.

**IMAGE 6:**
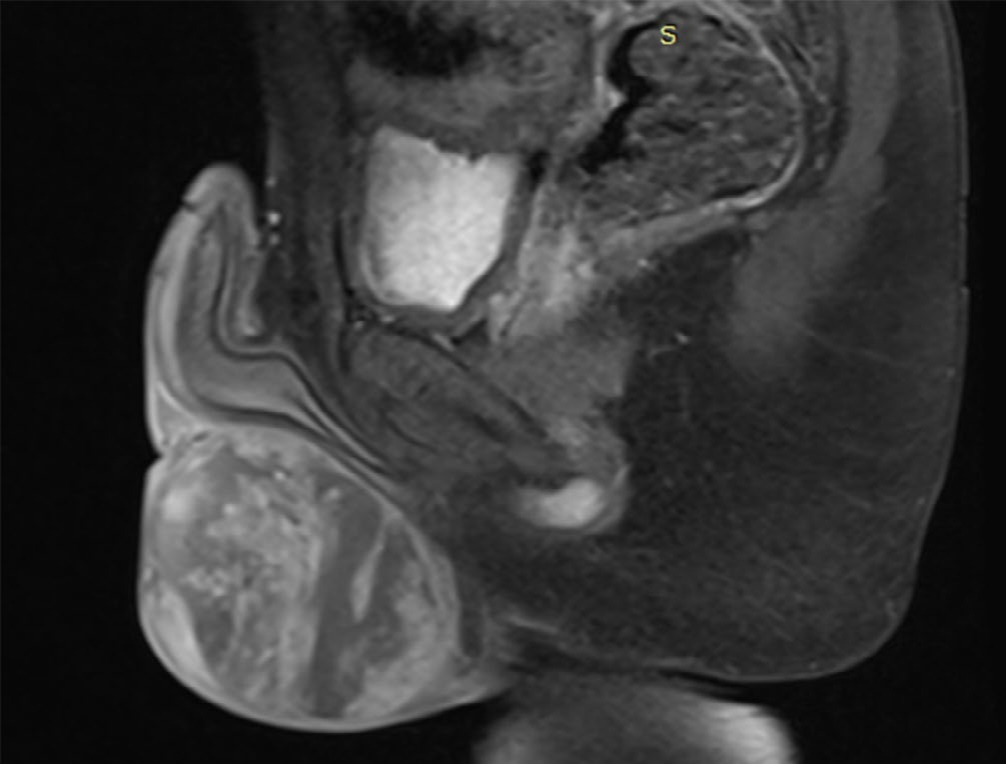
Sagittal T1‐weighted post‐contrast image: Following gadolinium administration, the lesion demonstrates heterogeneous enhancement, with more avid enhancement of the solid components and relatively non‐enhancing areas consistent with necrosis.

**IMAGE 7:**
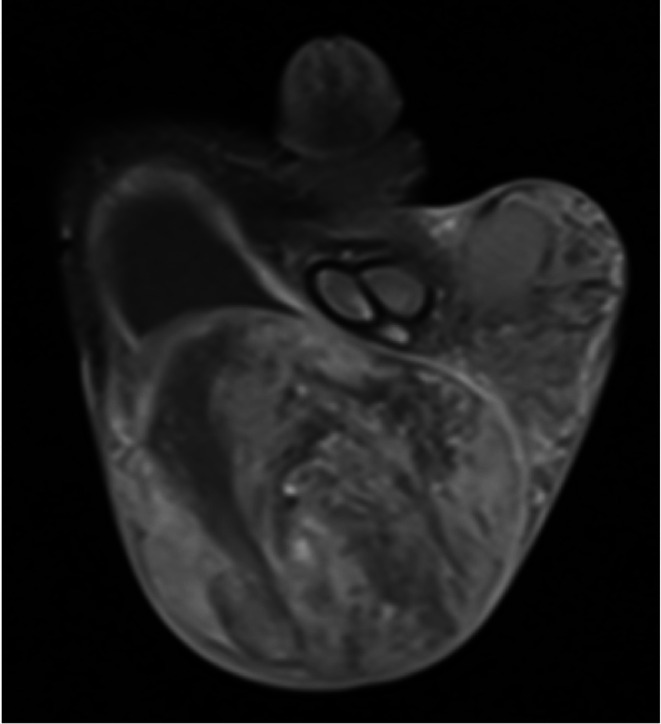
Coronal and axial T1‐weighted post‐contrast image: The enhancing soft‐tissue mass is clearly delineated from the right testicle, which shows normal homogeneous enhancement. The lesion demonstrates irregular but well‐defined margins, without definite invasion of the testicular parenchyma. Axial images confirm the paratesticular location, showing the mass adjacent to but distinct from the spermatic cord structures.

**IMAGE 8:**
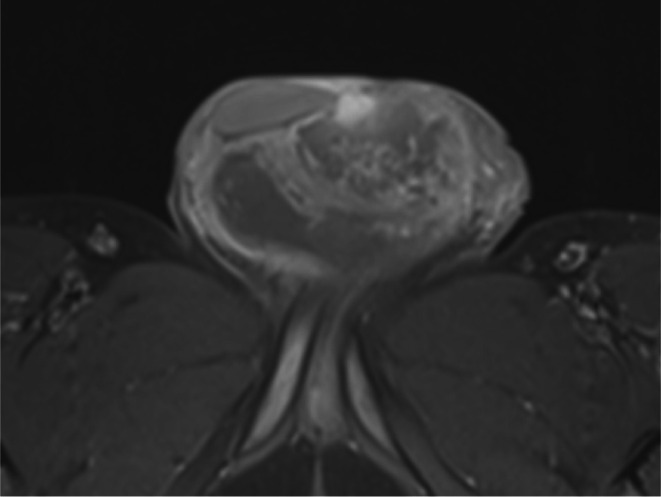
Coronal and axial T1‐weighted post‐contrast image which shows normal homogeneous enhancement. The lesion demonstrates irregular but well‐defined margins, without definite invasion of the testicular parenchyma. Axial images confirm the paratesticular location, showing the mass adjacent to but distinct from the spermatic cord structures.

Magnetic resonance imaging of the scrotum demonstrates a right paratesticular mass highly suspicious for malignancy. Coronal T2‐weighted image shows a lobulated, heterogeneous soft‐tissue mass centered in the right paratesticular region with intermediate to high T2 signal intensity (Image 3). Coronal T2‐weighted image demonstrates the right testicle (arrow) displaced inferiorly and medially but remaining separate and preserved with normal T2 signal (Image 4). Sagittal T1‐weighted pre‐contrast image shows the mass appearing iso‐ to mildly hypointense relative to skeletal muscle (Image 5). Sagittal T1‐weighted post‐contrast image reveals heterogeneous enhancement with solid components showing avid enhancement and areas of non‐enhancement (Image 6). Coronal and axial T1‐weighted post‐contrast images clearly delineate the enhancing paratesticular mass from the right testicle, confirming the paratesticular location without definite testicular invasion (Images 7 and 8).

For staging, CT of the chest, abdomen, and pelvis with intravenous contrast was performed. Chest CT showed normal bilateral lung fields without masses or nodules, clear mediastinum without lymphadenopathy, no pleural effusion, and no bone lesions. Abdominal and pelvic CT confirmed the 7.5 × 8.0 × 8.0 cm heterogeneously enhancing right scrotal mass extending into the inguinal canal with mixed soft tissue and cystic components, few prominent para‐aortic lymph nodes, and few prominent bilateral inguinal lymph nodes. The remaining abdominal and pelvic viscera were unremarkable with no evidence of metastatic disease. Following persistent clinical symptoms and imaging findings suspicious for malignancy, serum AFP, β‐hCG, LDH, and cross‐sectional staging imaging were obtained to exclude a germ cell tumor and guide definitive management. Tumor markers including AFP, β‐HCG, and LDH were negative, making germ cell tumor origin unlikely.

Given the imaging findings highly suspicious for a paratesticular malignancy and the failure of conservative management, the patient underwent right radical orchiectomy via an inguinal approach. After obtaining informed consent and under general anesthesia, a right inguinal incision was made with careful dissection to expose the spermatic cord at the level of the internal ring. The right testis was markedly enlarged and firm with an irregular surface. A large heterogeneous paratesticular mass involving the epididymis and extending into the inguinal canal was identified, with areas of cystic degeneration and necrosis. The tunica vaginalis was thickened with serosanguinous fluid present. The spermatic cord was isolated, clamped, ligated, and divided high at the internal inguinal ring to ensure oncological adequacy. The specimen consisting of the right testis, epididymis, paratesticular mass, and spermatic cord was delivered en bloc and sent for histopathological examination. Hemostasis was secured, and the wound was closed in anatomical layers. The patient tolerated the procedure well with minimal blood loss (< 50 mL) and was transferred to recovery in stable condition.

Gross pathological examination revealed a 10 × 8 × 6 cm specimen with a paratesticular mass measuring 8.5 × 8.5 × 6 cm. The mass was fragmented, lobulated, tan‐white, and firm with focal hemorrhage and necrosis. The tumor compressed the adjacent testis with focal infiltration. The tunica vaginalis was ruptured. The epididymis was involved by tumor, while the spermatic cord appeared unremarkable.

**IMAGE 9:**
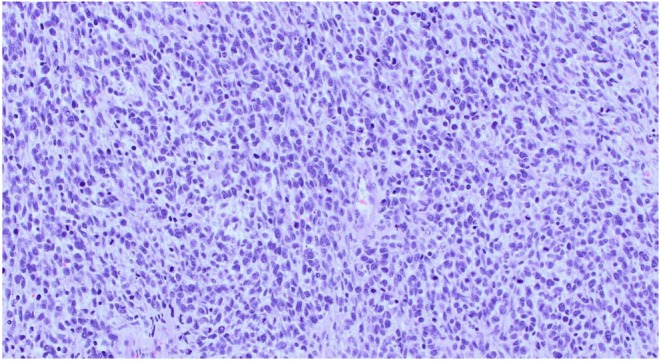
An ×10 cellular tumor composed of small round to spindled shape cells, some of them show rhabdomyoblastic differentiation (*strap cells*), with scant eosinophilic cytoplasm, arranged in intersecting fascicles.

**IMAGE 10:**
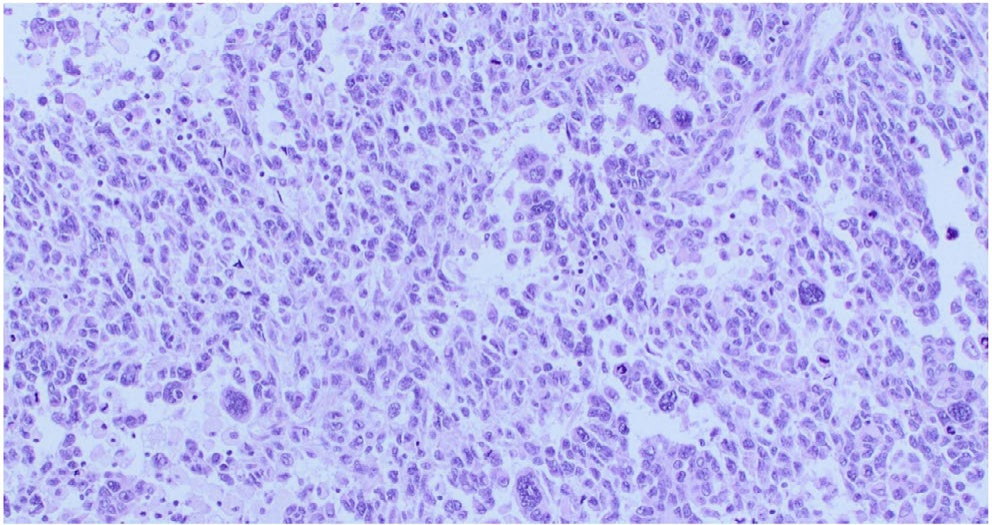
An ×20 cellular tumor composed of small round to spindled shape cells, some of them show rhabdomyoblastic differentiation (*strap cells*), with scant eosinophilic cytoplasm, arranged in intersecting fascicles.

Microscopic examination demonstrated a highly cellular tumor composed of small round to spindle‐shaped cells with rhabdomyoblastic differentiation (strap cells) arranged in intersecting fascicles. Perivascular condensation with brisk mitotic activity was observed (Image 9). Notably, focal regions within the tumor displayed increased cellularity and marked nuclear pleomorphism consistent with anaplastic features. These anaplastic areas showed rhabdomyoblasts with eccentric nuclei, prominent nucleoli, and abundant eosinophilic cytoplasm (Image 10). The stroma demonstrated loose myxoid changes characteristic of myogenic tumors.

**IMAGE 11:**
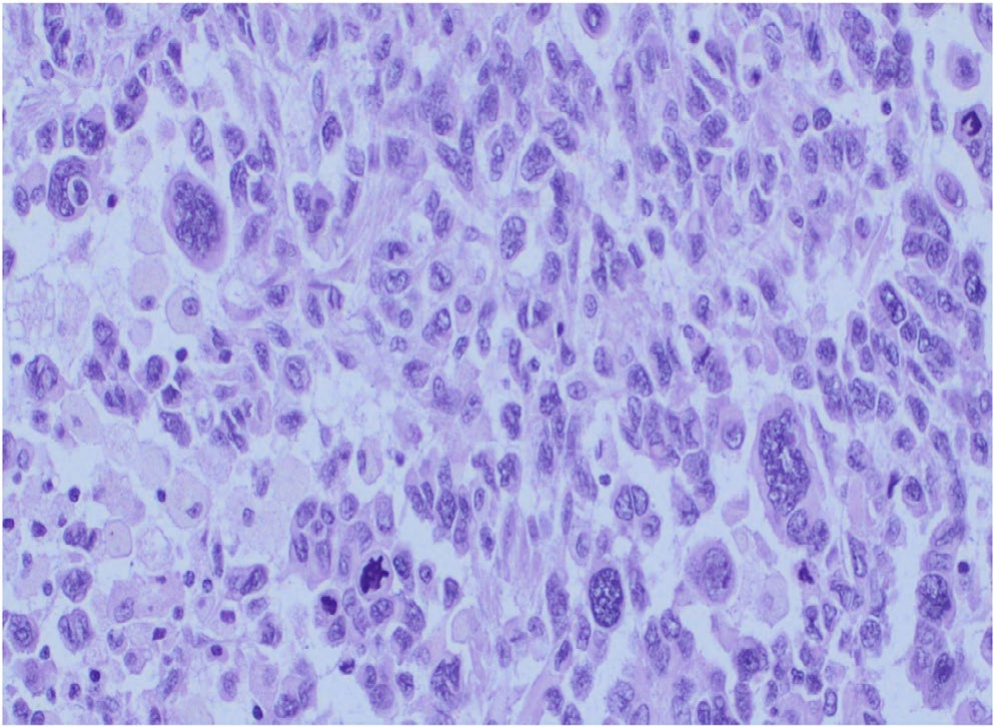
×20 Histopathological examination (hematoxylin and eosin staining) shows embryonal rhabdomyosarcoma with small round to spindle‐shaped cells demonstrating rhabdomyoblastic differentiation (strap cells) with scant eosinophilic cytoplasm, arranged in intersecting fascicles.

**IMAGE 12:**
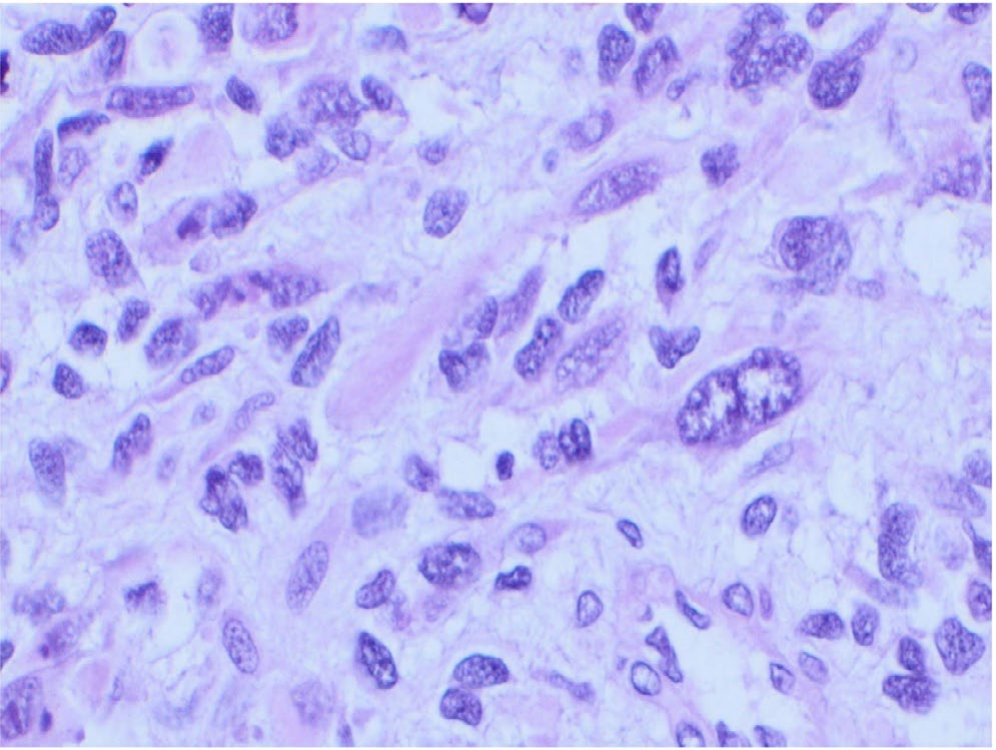
×40 Histopathological examination (hematoxylin and eosin staining) shows embryonal rhabdomyosarcoma with small round to spindle‐shaped cells demonstrating rhabdomyoblastic differentiation (strap cells) with scant eosinophilic cytoplasm, arranged in intersecting fascicles.

Histopathological examination (hematoxylin and eosin staining) shows embryonal RMS with small round to spindle‐shaped cells demonstrating rhabdomyoblastic differentiation (strap cells) with scant eosinophilic cytoplasm, arranged in intersecting fascicles (Images 11 and 12). 10× magnification showing overall tumor architecture (Image 9). 20× magnification demonstrating perivascular condensation and brisk mitotic activity within the fascicles (Image 11).

High‐power magnification of focal anaplastic regions within the tumor. 20× magnification showing focal anaplastic features with marked nuclear pleomorphism and cells displaying eccentric nuclei with prominent nucleoli (Image 11). 40× magnification demonstrating increased eosinophilic cytoplasm and heightened cellular atypia characteristic of anaplastic differentiation (Image 12).

**IMAGE 13:**
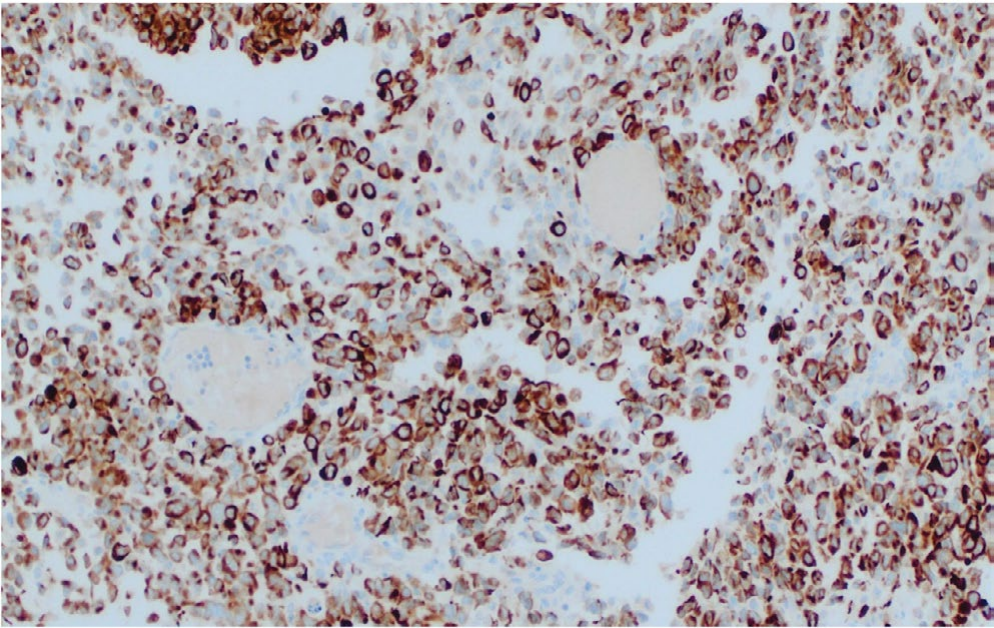
Desmin immunostain showing strong and diffuse positivity in tumor cells.

Immunohistochemical studies confirmed skeletal muscle origin: tumor cells were strongly positive for desmin and myogenin, consistent with myogenic differentiation, and negative for caldesmon and pan‐cytokeratin (PanCK), excluding smooth muscle and epithelial origins (Image 13).

**IMAGE 14:**
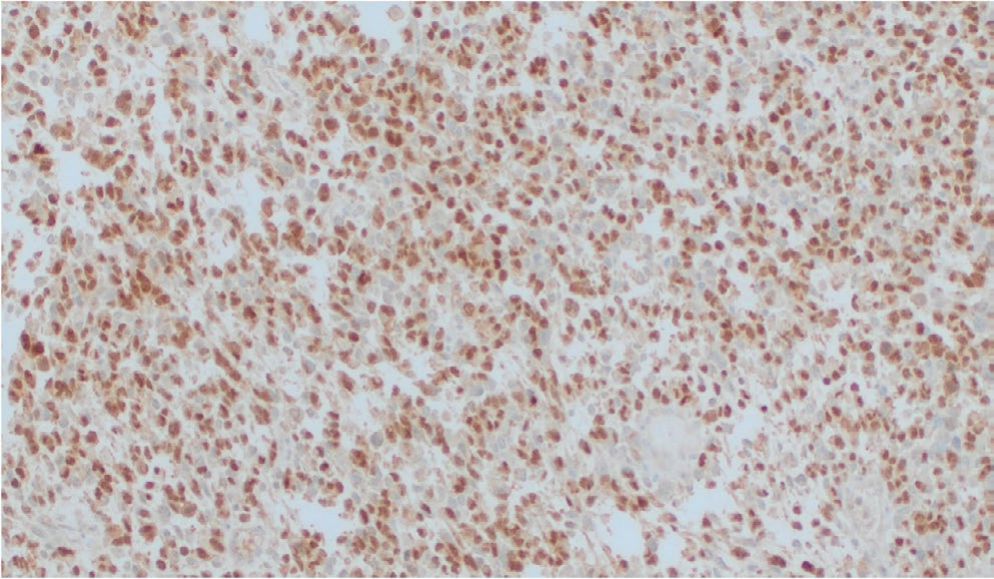
Myogenin immunostain demonstrating positive nuclear staining specific for myogenic lineage.

**IMAGE 15:**
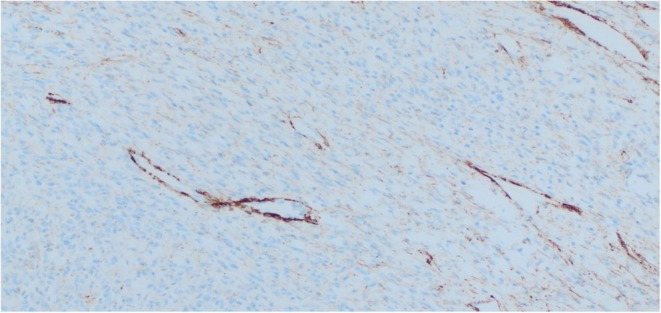
Caldesmon immunostain is negative, excluding smooth muscle origin.

**IMAGE 16:**
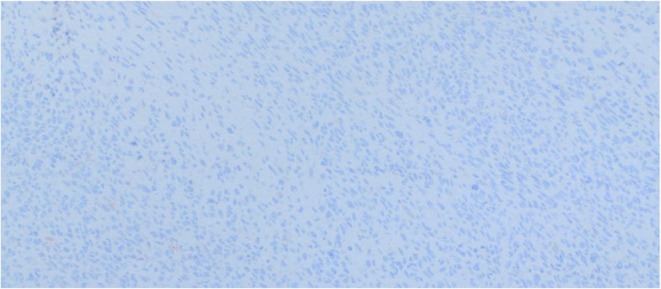
Pan‐cytokeratin (PanCK) immunostain is negative, excluding epithelial origin.

Immunohistochemical staining confirms skeletal muscle differentiation and excludes alternative diagnoses. Desmin immunostain showing strong and diffuse positivity in tumor cells (Image 13). Myogenin immunostain demonstrating positive nuclear staining specific for myogenic lineage (Image 14). Caldesmon immunostain is negative, excluding smooth muscle origin (Image 15). PanCK immunostain is negative, excluding epithelial origin (Image 16).

## 3 Differential Diagnosis

The differential diagnosis of scrotal masses in pediatric and adolescent patients is broad and includes both benign and malignant etiologies. Benign conditions encompass inflammatory lesions (epididymo‐orchitis, orchitis, testicular abscess), vascular pathology (testicular torsion, torsion of testicular appendages), developmental anomalies (hydrocele, varicocele, spermatocele), and hernias [7]. Among malignant etiologies, germ cell tumors predominate, particularly yolk sac tumors in prepubertal boys and mixed germ cell tumors in adolescents [8]. Paratesticular sarcomas, while uncommon, include RMS (most common), leiomyosarcoma, liposarcoma, fibrosarcoma, and malignant fibrous histiocytoma [9]. Distinguishing paratesticular RMS from these entities relies on careful correlation of clinical presentation, imaging characteristics, tumor markers (which are negative in RMS), and definitive histopathology demonstrating rhabdomyoblastic differentiation. The presence of desmin and myogenin immunopositivity is pathognomonic and distinguishes RMS from other sarcoma subtypes [10]. Recognition of atypical presentations, such as acute inflammatory features or failure to respond to antibiotic therapy, is crucial to avoid diagnostic delays and ensure appropriate oncological management.

## 4 Results and Follow‐Up

Postoperatively, the patient recovered without complications. He was referred to pediatric oncology for risk stratification and initiation of adjuvant chemotherapy according to COG protocols for high‐risk paratesticular RMS. At the 3‐month follow‐up, the patient was tolerating chemotherapy well, with no clinical evidence of local recurrence or distant disease progression.

## 5 Conclusion

Paratesticular embryonal RMS should be considered in adolescents presenting with painful scrotal swelling that fails to respond to appropriate antibiotic therapy, particularly when ultrasonography demonstrates a heterogeneous vascular paratesticular mass. This case highlights the importance of early reconsideration of the diagnosis, timely advanced imaging, and referral to a pediatric oncology center to avoid delays in definitive management. Early diagnosis and multidisciplinary treatment, including radical inguinal orchiectomy and risk‐adapted systemic therapy, remain essential for optimizing oncological outcomes. To our knowledge, this represents the first reported case of paratesticular embryonal RMS from Palestine, contributing to the limited literature on this rare pediatric malignancy and emphasizing the need for increased clinical awareness of its atypical inflammatory presentation.

## 6 Discussion

Paratesticular RMS is a rare and aggressive malignancy predominantly affecting children and adolescents [1]. While RMS is the most common soft tissue sarcoma in pediatrics, accounting for approximately 50% of soft tissue sarcomas in this age group, paratesticular RMS specifically represents only 7% of all RMS cases [1]. The embryonic origin of RMS lies in primitive mesenchymal cells committed to skeletal muscle differentiation [11]. During embryogenesis, myogenic regulatory factors including MyoD and myogenin drive the differentiation of these primitive cells toward skeletal muscle lineage [11]. When this developmental program becomes dysregulated, rhabdomyoblasts fail to properly differentiate and instead proliferate, resulting in RMS [11]. The paratesticular region, which includes the spermatic cord, epididymis, and testicular tunics, represents an uncommon primary site, with most RMSs arising in the head and neck (35%) or genitourinary tract (25%) [9].

Histologically, RMS is categorized into four main subtypes according to the WHO classification: embryonal (ERMS), alveolar (ARMS), pleomorphic, and spindle cell/sclerosing variants [8]. Embryonal RMS is the predominant histologic subtype in paratesticular disease, accounting for 90%–97% of cases, and is characterized by primitive round to spindle‐shaped cells with scant cytoplasm arranged in loose fascicles [7]. The presence of “strap cells” with abundant eosinophilic cytoplasm and eccentric nuclei is pathognomonic for rhabdomyoblastic differentiation [10]. In contrast, alveolar RMS, characterized by distinctive “alveolar” architecture with tumor cells lining fibrovascular septa, demonstrates more aggressive behavior with rapid progression and higher mortality rates [8].

Management of paratesticular RMS requires a multidisciplinary approach combining surgery, systemic chemotherapy, and selected use of radiotherapy. Radical inguinal orchiectomy remains the standard surgical treatment, while postoperative chemotherapy according to COG protocols significantly improves survival in localized disease. Radiotherapy is considered for patients with residual disease, nodal involvement, or other high‐risk features. Careful staging and multidisciplinary planning are therefore essential to optimize long‐term oncological outcomes [12].

According to Table 1, from 2012 to 2025, we identified three well‐documented pediatric and adolescent cases of paratesticular RMS in the recent literature, allowing comparison with the present case. Our review showed a male predominance (100%), which is consistent with the anatomical location. Most patients were symptomatic, with presentation ranging from painless masses to acute painful swelling. The tumor size ranged from 80 to 165 mm, with the current case (85 mm) falling in the mid‐range. Patient ages spanned from 6 to 16 years, with a median of 14.5 years, indicating a diagnostic concentration in adolescence.

**TABLE 1:** Reported cases of paratesticular rhabdomyosarcoma in pediatric and adolescent patients (2012–2025).

Author	Year	Sex	Age	Symptoms	Size	Histology	Key features	Therapy
Kim et al. [7]	2012	M	10	Painful scrotal swelling, 2 weeks	Unspecified	Embryonal	Misdiagnosed epididymitis	Orchiectomy + chemo
Shazly et al. [10]	2016	M	16	Painless paratesticular mass	80 mm	Embryonal (plastic variant) Grade III	No lymph node metastasis	Inguinal orchiectomy + chemo
Abuisneneh et al. [8]	2025	M	15	Painless post‐trauma, 7 months	150 × 110 × 100 mm	Embryonal	Incidental during trauma surgery	Orchiectomy + chemo + RT
Present case	2025	M	14	Acute pain (10/10), 1 month antibiotic failure	85 × 85 × 60 mm	Embryonal, spindle cell, focal anaplasia	Prolonged epididymitis misdiagnosis	Radical orchiectomy + VAC chemo

Tumor sizes ranged from 80 to 165 mm. The current case (85 mm) was among the smaller documented lesions. The largest, measuring 150 × 110 × 100 mm, was reported in 2025 in a 15‐year‐old patient who presented 7 months after testicular trauma [8]. Patient ages ranged from 6 to 16 years, with a median of 14.5 years. The majority of cases occurred in adolescents aged 14–16, indicating a diagnostic concentration in mid‐to‐late adolescence, a period when clinical suspicion for malignancy may be lower due to the relative rarity of testicular tumors in this age group [13].

The imaging characteristics of our case a large heterogeneous paratesticular mass with increased vascularity on Doppler ultrasound, heterogeneous T2 signal with areas of necrosis on MRI, and prominent regional lymph nodes on CT are highly suggestive of paratesticular RMS. However, what distinguishes this case is the initial presentation with acute inflammatory features including severe pain, scrotal skin thickening, and pyocele, which led to prolonged misdiagnosis as epididymo‐orchitis. This clinical presentation mimics benign infectious pathology and represents a well‐documented diagnostic pitfall that can result in significant treatment delays [7]. The persistence of symptoms despite 1 month of intensive antibiotic therapy, including intravenous meropenem, ultimately prompted advanced imaging. This prolonged course underscores the critical importance of maintaining high clinical suspicion when pediatric patients with scrotal masses fail to respond to appropriate antimicrobial therapy.

Although enlarged para‐aortic lymph nodes were identified on staging CT, retroperitoneal lymph node dissection was not performed. Following multidisciplinary discussion, the patient proceeded directly to systemic chemotherapy according to pediatric oncology protocols because management was based on radiological staging and planned multimodal therapy. Current COG protocols increasingly individualize the role of RPLND according to imaging findings, patient age, and response to systemic treatment.

An additional distinguishing feature of our case is the presence of focal anaplasia within embryonal RMS with spindle cell morphology. Anaplasia, characterized by marked nuclear enlargement, hyperchromasia, and atypical mitotic figures, has been a subject of considerable debate regarding its prognostic significance. The largest prospective study to date, involving 1648 patients enrolled in consecutive COG trials, found an overall prevalence of anaplasia of 19% (8% focal, 11% diffuse) in RMS [11]. Importantly, this landmark study demonstrated that anaplasia was not an independent prognostic factor when controlled for other established variables in multivariate analysis [11]. However, emerging molecular evidence suggests that anaplasia may serve as a surrogate marker for *TP53* mutations; in the COG study subset with available mutational data, *TP53* mutations were present in 24% of anaplastic tumors, while 69% of tumors with *TP53* mutations demonstrated anaplastic morphology [11]. These findings suggest that while anaplasia itself may not independently determine prognosis, it should prompt molecular testing for *TP53* mutations, which are independently associated with inferior outcomes and increased radio resistance.

## Author Contributions

**Saif Khaled Abdalhadi Azzam:** writing – original draft. **Lina Barhoum Barhoum:** data curation. **Ammir Abuzahra:** visualization, writing – review and editing. **Alaa Alazzeh:** writing – review and editing. **Hamza Zughaier:** project administration. **Khader Hassouneh:** writing – review and editing. **Nour Aldin K. Fakhory:** software. **Ibrahim Alzatari:** data curation. **M. I. Jawabreh Issa:** methodology. **Mohd Othman Lafi:** validation.

## Funding

The authors have nothing to report. The authors voluntarily contributed to this case report without external or institutional funding.

## Ethics Statement

Ethical approval for publication of this case was obtained from the Ethics Committee of Al‐Ahli hospital, with documentation available for editorial review upon request.

## Consent

Written informed consent was obtained from the patient for publication of this case report. A copy of the signed consent form is retained by the authors and is available for editorial review upon request.

## Conflicts of Interest

The authors declare no conflicts of interest.

## Data Availability

Data are available on request due to privacy/ethical restrictions.
